# Inequality signals in dorsolateral prefrontal cortex inform social preference models

**DOI:** 10.1093/scan/nsy020

**Published:** 2018-04-04

**Authors:** Lisa Holper, Christopher J Burke, Christoph Fausch, Erich Seifritz, Philippe N Tobler

**Affiliations:** 1 Department of Psychiatry, Psychotherapy, and Psychosomatics, University Hospital of Psychiatry Zurich, 8032 Zurich, Switzerland; 2 Laboratory for Social and Neural Systems Research, Department of Economics, University of Zurich, 8006 Zurich, Switzerland

**Keywords:** ultimatum game, inequity aversion, punishment, decision neuroscience, functional near-infrared spectroscopy

## Abstract

Humans typically display inequality aversion in social situations, which manifests itself as a preference for fairer distributions of resources. However, people differ in the degree to which they dislike being worse off [disadvantageous inequality (DI) aversion] or better off [advantageous inequality (AI) aversion] than others. Competing models explain such behavior by focusing on aversion to payoff differences, maximization of total payoff or reciprocity. Using functional near-infrared spectroscopy, we asked which of these theories could better explain dorsolateral prefrontal cortex (dlPFC) activity while participants accepted or punished fair *vs* unfair monetary transfers in an anonymous norm compliance task. We found that while all participants exhibited DI aversion, there were substantial differences in preferences for AI, which were strongly predicted by dlPFC activation. Model comparisons revealed that both punishment behavior and prefrontal activity were best explained by a model that allowed for AI seeking rather than imposing aversion. Moreover, enhancing this model by taking into account behavioral response times, as a proxy for choice difficulty, further improved model fits. Our data provide evidence that the dlPFC encodes subjective values of payoff inequality and that this representation is richer than envisaged by standard models of social preferences.

## Introduction

Social preferences and inequality aversion have been incorporated into modern economic theories to capture the fact that humans are not only concerned about their own payoffs but also about the relation between their own payoffs and those of others. These payoff differences can take two forms depending on how they are distributed. Disadvantageous inequality (DI) occurs when one receives less than others and advantageous inequality (AI) occurs when one receives more than others (Walster *et al.*, 1978; Fehr and Schmidt, 1999). Behavioral experiments show that people are averse to both types of inequality to differing degrees (Fehr and Schmidt, 1999). In general, they are willing to pay more to avoid DI than AI, suggesting that DI is more aversive than AI (Loewenstein *et al.*, 1989; Fehr and Schmidt, 1999; Dawes *et al.*, 2007). However, it is also possible that some individuals may gain satisfaction from earning more than others. These individuals may actively seek AI by accepting a cost to reduce the payoffs of others (Vostroknutov *et al.*, 2012).

In behavioral economics, models are used to specify the relation between preferences and observable behavior. There are several competing economic models that seek to explain how social preferences and inequality aversion relate to behavior. Essentially these models were developed to explain behavior that could not be accounted for by standard economic theories which consider only self-interest as an economically relevant preference. For example, standard economic theory suggests that when deciding whether to accept or reject a proposed distribution of money (i.e. an offer), responders should and will always accept any non-zero offer in the standard ultimatum game as it leaves them better off than rejecting it. Contrary to this prediction, responders regularly reject unfair offers so that both players receive nothing (Güth *et al.*, 1982), a behavior that can be explained by non-standard economic models that incorporate social preferences.

These models take a number of different forms with the simplest consisting of ‘difference aversion’ models (e.g., Fehr and Schmidt, 1999) where fairness considerations motivate people to reduce differences between their own and others’ payoffs. In contrast, ‘efficiency’ models (e.g., Charness and Rabin, 2002) assume that people are motivated to maximize the total payoff of all parties. Moreover, Charness and Rabin (2002) propose that people increase or decrease the payoffs of others depending on perceived fairness of the behavior of these others (reciprocity). While both types of models account for subjective preferences regarding both DI and AI, it remains largely unknown whether they explain behavior and neural activity equally well and to what degree their restrictive assumptions are realistic. In particular, the strict assumptions of difference aversion models do not allow for AI-seeking behavior and that DI aversion may be weaker than AI aversion. We aimed to address this issue by allowing participants to engage in AI-seeking behavior and comparing unconstrained instances of difference aversion (Fehr and Schmidt, 1999) against efficiency models (Charness and Rabin, 2002), while recording neural activity using functional near-infrared spectroscopy (fNIRS).

Previous neuroimaging studies investigating neural responses to DI and AI suggest the dorsolateral prefrontal cortex (dlPFC) to be particularly involved in encoding and interpreting payoff inequalities and implementing inequality averse behaviors (Sanfey *et al.*, 2003; Hsu *et al.*, 2005; Haruno and Frith, 2010; Tricomi *et al.*, 2010; Chang *et al.*, 2011; Fliessbach *et al.*, 2012; Cappelen *et al.*, 2014; Güroğlu *et al.*, 2014; Haruno *et al.*, 2014; Yu *et al.*, 2014; Nihonsugi *et al.*, 2015; ). Examples include rejecting unfair offers in the ultimatum game (Knoch *et al.*, 2006) and enforcing social norm compliance (Ruff *et al.*, 2013). These findings suggest that the dlPFC encodes social utility signals that reflect the social preferences of a given individual. However, this hypothesis remains to be tested and at the neural level, candidates for social utility signals have rarely been characterized with formal models from economic theory.

**Fig. 1.**
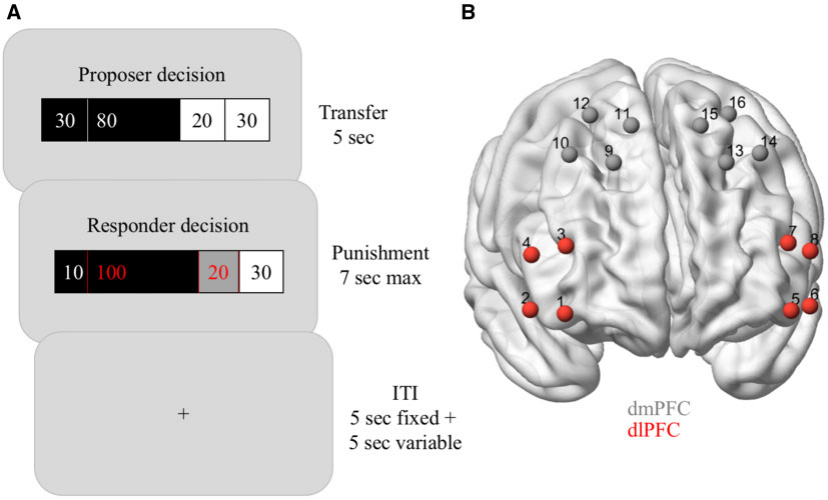
Experimental design. (A) Trial timeline. Top, proposer decision. Each trial started with a 5-s screen that informed the responder (the participant) about the constant initial endowment of both players (30 points, displayed at both ends) and about the proposer’s transfer (white number on black background indicates the amount kept by the proposer, black number on white background indicates the proposed transfer). Here, the proposer has offered a transfer of 20 points, leaving the proposer at this stage with 110 points in total and the responder with 50 points (i.e. the responder is in a state of DI). Middle, responder decision. The responder then had 7 s to accept the transfer (such that the proposer would be left with 110 points and the responder with 50 points) or to punish the proposer by spending points from the initial endowment of 30 points. Red numbers on dark backgrounds indicate the points that will be removed from the proposer (black background) and the responder (dark gray background) as a result of the punishment decision (Table 1). In this case, the responder has chosen to spend 20 points to reduce the proposer’s payoff by 100 points. This leaves the proposer with 10 and the responder with 30 points (i.e. the responder is now in a state of AI). Bottom, trials were separated by an inter-trial-interval which lasted 5 s plus a uniformly distributed jitter with a mean duration of 5 s. (B) fNIRS channel positions. Channel positions covering the dlPFC (channels 1–8) and the dorsomedial prefrontal cortex (dmPFC, channels 9–16).

To close this gap, we recorded prefrontal activity using fNIRS while participants performed a modified version of a norm compliance task (Spitzer *et al.*, 2007; Ruff *et al.*, 2013) (Figure 1A). In this task, the participant (the responder) received clearly fair to extremely unfair transfers from another, anonymous, person (the proposer). Participants could either accept the transfer as it was, or they could punish the proposer (i.e. reduce the proposer’s payoff) by using points from their own endowment. Crucially, this allowed participants to create advantageous, equal or disadvantageous final payoff distributions according to their social preferences, thereby giving them a wider range of behaviors to choose from than in previous investigations into social preferences. In particular, our task allowed participants to engage in AI-seeking behavior (but not AI-averse behavior), thus allowing us to test the assumptions and constraints of previous social preference models.

To characterize behavioral and prefrontal responses during this task we compared the unconstrained Fehr–Schmidt model and Charness–Rabin model with several alternative models (Báez-Mendoza *et al.*, 2016). We also aimed to investigate whether a model that included behavioral response time (RT) would explain additional variance compared to the winning model (Báez-Mendoza *et al.*, 2016), because previous work reported that RT correlates with prefrontal activity (Yarkoni *et al.*, 2009; Grinband *et al.*, 2011) and is sensitive to reward inequality (Fischbacher *et al.*, 2013; Báez-Mendoza *et al.*, 2016). Accordingly, we expected that the addition of RT as parameter in the winning model would improve model fit for both behavioral and neural responses.

## Materials and methods

### Participants

Forty-eight healthy right-handed participants (28 females, age 23 ± 2.3, mean ± s.d.) were recruited at the University of Zurich. Exclusion criteria were any history of psychiatric or neurological disorders or current medication. All participants gave written informed consent. The study was approved by the ethics committee of the Canton Zurich.

### Experimental protocol

We investigated the behavioral and neural reactions of responders to pre-recorded fair *vs* unfair transfers from anonymous proposers. We used a variant of the ultimatum game known as the norm-compliance task in which a proposer makes an offer to split an initial endowment between themselves and the responder (i.e. our participants). After this offer is made, participants could, at a cost to themselves, reduce the payoff of the proposer (i.e. punish them) and the level of punishment could be varied to produce different levels of inequality (Spitzer *et al.*, 2007; Ruff *et al.*, 2013). We used offers from proposers that were collected from a previous experiment and instructed participants that they would be responding to these in a non-repeating and anonymous fashion. Accordingly, all interactions with any given proposer were limited to one response. All participants saw the same distribution of offers but in a randomized order (Figure 1A).

On every trial, both players received an initial endowment of 30 points (Table 1). The proposer additionally received another 100 points that he or she could share with the responder by making an offer of 0, 10, 20, 30, 40 or 50 points (i.e. offers ranged from fair**—**an equal split of 50 points**—**to very unfair—offering 0 points). After viewing the offer of the proposer, the responder had the option to accept the transfer or to use a portion or all of their initial endowment of 30 points to punish the proposer. For every point that the responder spent on punishment, the proposer’s payoffs were reduced by five points, creating a substantial leveraging effect. This means for example, that if the proposer transferred 20 out of 100 points to the responder such that after the transfer decision the proposer had 110 points (80 out of 100 points** **+ 30 from the initial endowment) and the responder had 50 points (20 out of 100 points** **+ 30 from the initial endowment), the responder could reduce the proposer’s payoff to 0 (and thereby create AI at the expense of responder payoff and efficiency) by paying 22 points of the initial endowment to punish the proposer for their unfair offer. Responders could vary how much endowment they spent on punishment by moving the mouse left or right (the initial starting point was randomly determined) and view how the payoffs of both the proposer and themselves would change. A final decision was made by the responder clicking the mouse button at their desired level of punishment. Trials were separated by intertrial intervals of 5 s (fixed)** **+ 5 s (univariate jitter).

**Table 1.** Possible behavior in experimental task

	Proposer (pre-recorded)	Responders (participants)
Initial endowment (fixed)	30 + 100	30
Transfer (in steps of 10)	0–50	
Punishment (in steps of 1)		0–30
Payoff (possible range)	0–130	0–80

Listed are the initial endowments, the possible punishments and payoffs. For every point that the responder invested into punishment, the proposer’s payoffs were reduced by five points.

All decisions were incentive compatible, as the points gained by the participants were transformed to Swiss Francs (CHF) after the experiment according to a predefined conversion rate (100 points** **= 1 CHF). These payoffs were paid out on top of a show up fee of 13 CHF. Prior to the experimental session, participants performed nine practice trials (not included in the analysis) to familiarize themselves with the task. During the main task, each participant completed 60 trials with an average duration of 19.6 ± 1.8 min.

### fNIRS instrumentation

fNIRS recordings were conducted using a NIRSport instrument (LLC NIRx Medical Technologies). The system utilized time-multiplexed dual-wavelength light-emitting diodes, each containing two light sources with wavelengths of 760 and 850 nm. Optical detection was performed using photo-electrical detectors containing Silicon photodiodes (Siemens, Germany). Sources and detectors were placed in a head cap to allow for direct skin contact (Epitex Inc., Japan). The data acquisition board was connected to a notebook computer running LabVIEW 2011 (National Instruments, Austin, TX, USA). The overall sampling rate was 10 Hz. The source-detector distance was ∼30 mm, with 16 channels covering parts of the dlPFC and the dorsomedial PFC (dmPFC; Figure 1B). Our hypotheses concerned primarily dlPFC and we therefore primarily present results from dlPFC. However, for completeness we also present comparisons between dlPFC and dmPFC (sections Neural model fit and dlPFC predicts behavior better than dmPFC). There were no significant differences between hemispheres (all *P* > 0.05) and we therefore averaged across both.

Functional recordings were pre-processed, including data detrending, filtering, baseline correction and motion artifact removal using the NIRSLab analysis software (Xu *et al.*, 2014). For baseline correction, the first 60 s of raw data were taken and subtracted from all channels. Motion artifacts (in particular, ‘steps’ and ‘spikes’) were removed in 10 participants after visual inspection. Hemodynamic concentration changes of [O_2_Hb] and [HHb] were calculated according to the Beer–Lambert law (Kocsis *et al.*, 2006) **[**absorption coefficients (µa) for O_2_Hb: µa(760 nm)** **= 1486, µa(850 nm)** **= 2526, for HHb: µa(760 nm)** **= 3843, µa(850 nm)** **= 1798; differential pathlength factor (DPF): DPF(760 nm)** **= 7.25, DPF(850 nm)** **= 6.38]. Total hemoglobin [tHb] derived as the sum of [O_2_Hb] and [HHb], served as primary parameter of interest because it represents changes in blood volume correlated with changes in blood flow (Grubb *et al.*, 1974). As [tHb] is thought to be far less sensitive to vein contamination, it provides higher spatial specificity for mapping cerebral activity compared to [O_2_Hb] or [HHb] separately (Gagnon *et al.*, 2012).

Δ[tHb] estimates were computed as a proxy for the underlying neural activity using the general linear model approach (Hoshi, 2016). The neural data were analyzed from the onset of the transfer by the proposer (transfer phase) to the end of the response by the participant (punishment phase). To determine the temporal profile of the hemodynamic response regarding model fit with higher temporal resolution, we chose a overlapping sliding window analysis, window width of 2.5 s, stepped every 180** **ms. Together the intervals covered 12 s of the hemodynamic response to both the transfer and the punishment phase.

### Statistical analysis

#### Models

The behavioral and neural data were fitted to various social preference models. First, we considered variants of the linear model of inequality aversion suggested by Fehr and Schmidt (1999). In the Fehr–Schmidt model, α and β are the inequality aversion parameters with α measuring aversion against DI, and β measuring aversion against AI. In their original model, Fehr and Schmidt (1999) imposed two constraints, that α ≥ β and that 0 ≤ β < 1. The first constraint α ≥ β captures the notion that a player suffers more from DI than AI. The second constraint 0 ≤ β < 1 rules out participants who like to be better off than others, and was imposed by Fehr and Schmidt (1999) although they noted that AI-seeking (β < 0) exists. To evaluate the adequacy of the imposed constraints, we tested the model once without (Unconstrained FS) and once with constraints (Standard FS). For the two-player case, the models correspond to:

**Fehr–Schmidt model *unconstrained* (Unconstrained FS):**

(1) $$U_{responder}={P_{reward\;responder}\;*\;X}_{responder}-\;\alpha \;*\;\max\left(X_{proposer}-X_{responder},\;0\right)-\;\beta \;*\;max(X_{responder}-X_{proposer},\;0)$$

*without* constraints on α and β

**Fehr–Schmidt model *constrained* (Standard FS):**

(2) As (1) but  with  constraints:α ≥ β  and 0  ≤ β < 1

The dependent variable, *U*_responder_, denotes utility. Here and in all the models described below, we replaced this variable with behavioral punishment amount for modeling of behavioral data or dlPFC activity for modeling of neural data (Báez-Mendoza *et al.*, 2016). *X*_proposer_ and *X*_responder_ are the post-punishment payoffs of the responder and proposer (both in units of points). *P*_reward responder_ is a parameter determining sensitivity of the participant to his/her own reward. While both models posit that the payoff difference determines social preferences, the unconstrained model allows for inequality seeking rather than only inequality aversion.

It is conceivable that dlPFC preferentially codes specific constituents of utility rather than a full social preference signal. Besides the Fehr–Schmidt model, we therefore also examined simpler and intermediate models (Báez-Mendoza *et al.*, 2016) that considered various combinations of the responder’s and proposer’s reward:

**Total Reward model:**

(3) $$U_{responder}=\;P_{reward\;total}\;*\;{(X}_{responder}+X_{proposer})$$

**Reward Difference model:**

(4) $$U_{responder}=\;P_{reward\;difference}\;*\;{(X}_{responder}-X_{proposer})$$

**Proposer Reward model:**

(5) $$U_{responder}=\;P_{reward\;proposer}\;*\;X_{proposer}$$

**Linear combination of responder and proposer reward:**

(6) $$U_{responder}=\;P_{reward\;responder\;}*\;X_{responder}+P_{reward\;proposer}\;*\;X_{proposer}$$

For comparison with the difference sensitivity models we tested the Charness–Rabin efficiency/reciprocity model (Charness and Rabin, 2002):

**Charness–Rabin model:**

(7) $$U_{responder}={(\rho r+\;\sigma s+\;\theta q)\;*\;X}_{proposer}+\;{(1-\rho r-\sigma s-\;\theta q)\;*\;X}_{responder}$$

where *r* = 1 if *X*_responder_ > *X*_proposer_ (i.e. AI), and *r* = 0 otherwise; *s* = 1 if *X*_responder_ < *X*_proposer_ (DI), and *s* = 0 otherwise; *q* = −1 if the proposer has misbehaved (which we defined as any transfer less than 50 points), and *q* = 0 otherwise. Here, the responder’s utility is a weighted sum of own and proposer payoff, with the weight depending on whether the proposer has behaved unfairly and on whether the responder is experiencing AI (weighted by ρ) or DI (weighted by σ). The relevance of efficiency is captured by utility increasing with the payoff of both players. The parameter θ provides a mechanism for modeling reciprocity, whereas the parameters σ and ρ rely solely on the payoffs and not on any notion of reciprocity. We also considered the three possible two-parameter variants of the Charness–Rabin model but none of these models fitted the data better than the full model and we therefore do not present them in detail.

Finally, we included responder RT as an additional parameter in the winning model. The rationale for this was that RT has been shown to correlate with prefrontal activity (Yarkoni *et al.*, 2009; Grinband *et al.*, 2011) and to be sensitive to reward inequality (Fischbacher *et al.*, 2013; Báez-Mendoza *et al.*, 2016). Since the winning model was Unconstrained FS in our analysis (see Results section), we tested whether the addition of the RT parameter would provide an even better fit:

**RT-enhanced Fehr–Schmidt model (** **Báez-Mendoza *et al.*,** 2016 **):**

(8) $$U_{responder}={P_{reward\;responder}\;*\;X}_{responder}-\;\alpha \;*\;\max\left(X_{proposer}-X_{responder},\;0\right)-\;\beta \;*\;\max\left(X_{responder}-X_{proposer},\;0\right)-\;P_{RT}\;*\;RT$$

Moreover, to assess how well the RT parameter alone would fit the data, we tested a RT-only model.

**RT-only model:**

(9) $$U_{responder}=P_{RT}\;*\;RT$$

The unconstrained Fehr–Schmidt model (as well as the RT-only model) is nested in the RT-enhanced Fehr–Schmidt model because the latter contains one (three) additional term(s). We therefore used a nested *F*-test to test whether RT-enhanced FS provided a better fit to the data than Unconstrained FS (or RT-only).

#### Behavioral model fit

For the behavioral data, we estimated the participant-specific best fitting parameters for each of the nine models using an optimization method for nonlinear least-squares (NLS) as implemented in MATLAB with lsqcurvefit. Multiple start locations were used to reduce the likelihood of the optimization algorithm getting stuck in local minima.

In the NLS procedure, we modeled the amount of behavioral punishment (dependent variable) with all models. To allow for comparison with the neural data, we standardized both the punishment data and neural data to values between −1 and 1.

For the unconstrained Fehr–Schmidt model, we did not impose the optimization constraints of the original model in the NLS, thereby allowing for punishment behavior not envisaged by the original model (e.g. AI-seeking behavior). In contrast, for Standard FS, we implemented the optimization constraints in the NLS as suggested by the original Fehr–Schmidt model.

To compare the goodness of fit of the various models, we used Akaike’s information criterion (AIC). The AIC provides an information theoretic basis for model comparison that considers both goodness of fit and parsimony, with smaller values indicating better fit to the data (Akaike, 1974). To examine the predictive power of the models, the mean-squared error (MSE) was calculated for each model. For statistical comparison of the MSEs, we added an approximation of a null-model in the form of randomly generated samples for each individual based on a uniform distribution. Statistical comparison of the MSEs was then performed using one-way ANOVA with Bonferroni correction to account for multiple comparisons.

#### Neural model fit

For the neural data (i.e. the hemodynamic responses), we estimated the participant-specific best fitting parameters for each of the nine models using multiple linear regression with robust fitting as implemented in MATLAB with fitlm and the ′fair′ weight function. As the fit of Standard FS to the behavioral data was clearly worse than that of any other model (Figure 3A), we did not consider it for the neural data. The regression was performed for each channel and each of the 40 sliding time intervals.

We modeled the hemodynamic data of each channel (dependent variable) with each of the models described above. Again, to allow for comparison with the behavioral data, we standardized the hemodynamic data to values between −1 and 1. To compare the goodness**-**of**-**fit of the various models, we applied AIC on MSE as for the behavioral data.

#### Social preference parameters

To compare the social preference parameters obtained from the winning behavioral model with those of the winning neural model (in both cases the RT-enhanced Fehr–Schmidt model), we performed a two-way ANOVA with the factors ‘data type’ (behavioral, neural) and ‘model parameters’ (*P*_reward responder_, α, β, *P_RT_*). Bonferroni correction was applied to account for multiple comparisons for the factor ‘model parameters’. To correlate behavioral and neural model parameters, we used percentage-bend correlation (Pernet *et al.*, 2012). This is a robust method that protects against outliers among marginal distributions and that may therefore provide better estimates of the true relationship between the model parameters (Rousselet and Pernet, 2012). We applied the default bending constant (0.2).

## Results

### Behavioral data

Responders punished the proposer to some degree in 80.5% of all trials even though punishment was costly for themselves, with 46 of 48 participants (96%) punishing at least once. These findings suggest that participants were motivated not only by maximizing their own payoffs. More specifically, they took advantage of the possibility to punish in order to both reduce DI (in line with DI aversion) and create AI (corresponding to AI seeking). Indeed, 38 participants (79%) used punishment to create AI at least once, even though doing so considerably reduced total payoffs and was particularly costly (see below). Together, participants appear to have been motivated not only by their own payoff but also by social preferences.

**Fig. 2.**
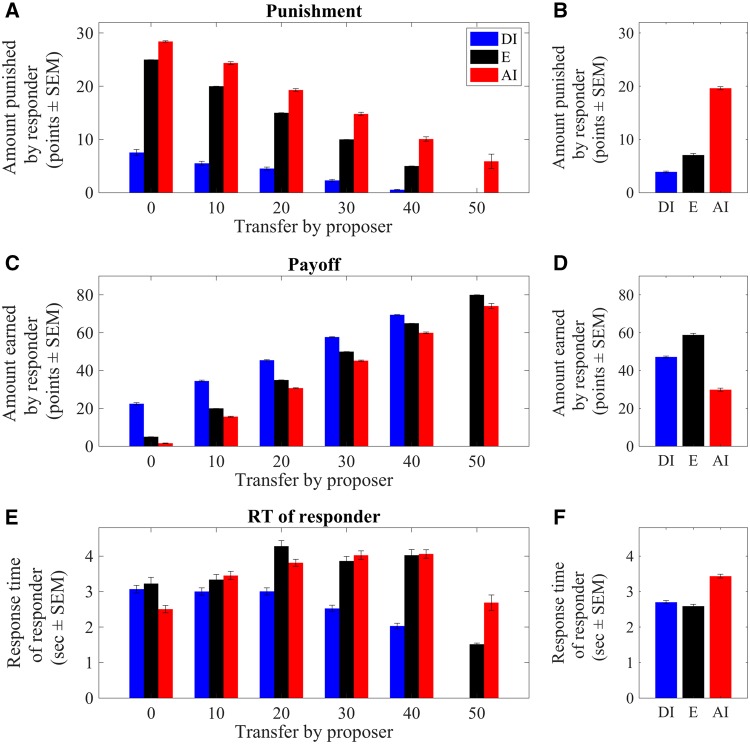
Behavioral data. (A) Responders punished the proposer less as transfer amounts of the proposer increased. (B) On average, in trials in which responders created AI they punished the proposer significantly more than in trials that resulted in *E* or DI. (C) Responders’ payoffs increased as transfers increased. (D) On average, given high transfer amounts combined with low punishment amounts, trials that resulted in *E* were associated with higher payoffs than those that resulted in AI or DI. (E) RTs of responders were highest at intermediate transfers of the proposer. (F) On average, RTs were higher for trials that resulted in AI and, to a lesser degree, DI compared to those that resulted in *E*. Error bars indicate standard error of the mean (SEM).

Decreasing transfers from fair (50 points) to unfair (0 points) significantly increased punishment amounts (Figure 2A, two-way ANOVA, main effect of ‘transfer’: *F*_5_ = 1117.91, *P* < 0.001). As expected given the structure of the task, creating AI was associated with significantly higher punishments than creating equality (*E*; *P* < 0.001) or DI (*P* < 0.001). Moreover, punishments were also higher for creating *E* than DI (*P* < 0.001) (Figure 2B, main effect of ‘inequality type’: *F*_2_ = 4001.83, *P* < 0.001; interaction of ‘transfer × inequality type’: *F*_9_ = 83.71, *P* < 0.001). Next, we considered the possibility that participants made more punishment decisions in the earlier phase of the task because they (incorrectly) assumed that they were participating in a repeated interaction game and thus could induce fairer offers on later trials. To test this possibility, we directly compared punishment levels between the first and second half of all trials. Contrary to the prediction, participants punished less in the first than the second half of trials, although this analysis only approached significance (*F* = 3.881, *P* = 0.049).

The lower punishments associated with higher transfers were also associated with significantly larger payoffs (Figure 2C; main effect of ‘transfer’: *F*_5_ = 13453.58, *P* < 0.001). Accordingly, responders earned most when proposers created *E* through fair transfers and responders in turn did not create AI (payoff difference between *E* and AI *P* < 0.001); with DI-tolerating final distributions, payoffs were significantly smaller than with final *E* (*P* < 0.001) but significantly higher than when participants created AI (*P* < 0.001) (Figure 2D, main effect of ‘inequality type’: *F*_2_ = 4001.83, *P* < 0.001; interaction effect of ‘transfer × inequality type’: *F*_9_ = 83.71, *P* < 0.001). Given the high costs of creating AI, it may appear all the more surprising that at least some participants were willing to incur these costs.

Next, we assessed RTs as a function of transfer size. Descriptively, RT showed a nonlinear quadratic pattern, i.e. responses were longest for intermediately unfair transfers and were faster, particularly for fair transfers but also for highly unfair transfers (Figure 2E; quadratic effect of ‘transfer’: *t*_5_ = −21.09, *P* < 0.001). This result can be explained in terms of decision difficulty; extremely unfair and fair offers are relatively easy to respond to (punish heavily or accept, respectively) whereas for intermediate levels of initial inequality the participant may be unsure as to how much to punish. Furthermore, responders required significantly more time in trials that resulted in AI or DI than in those resulting in *E* (Figure 2F; linear effect of ‘inequality type’: *t*_2_ = 3.54, *P* < 0.001, quadratic effect of ‘inequality type’: *t*_2_ = −4.18, *P* < 0.001). Thus, also RT reflected sensitivity to transfer amounts and inequality, with intermediately unfair transfers corresponding to the most difficult decisions.

### Behavioral model fit

**Fig. 3.**
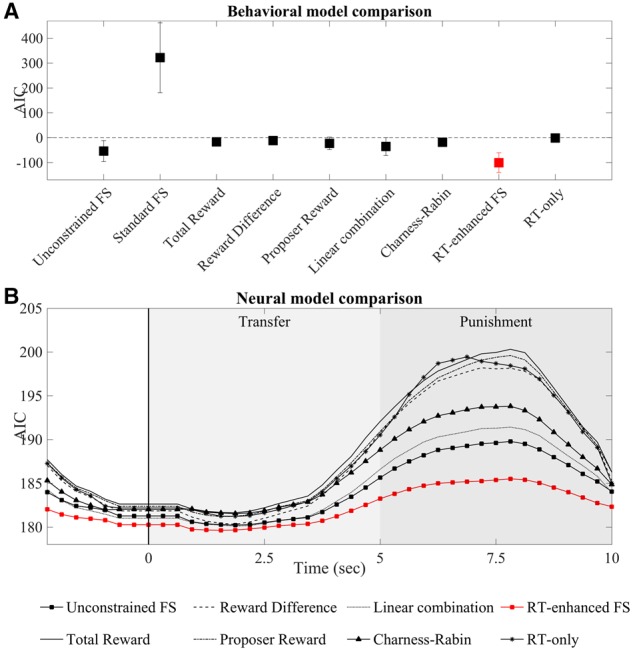
Behavioral and neural model comparisons. (A) Model AICs for punishment behavior. Smaller (more negative) values correspond to a relatively better fit of the corresponding model. The unconstrained Fehr–Schmidt model (Unconstrained FS) fitted punishment behavior consistently better compared to its constrained version (Standard FS), the simpler models (Total Reward, Reward Difference, Proposer Reward, Linear Combination), the Charness–Rabin model, and the RT-only model. Error bars represent standard deviation. (B) Model AICs for dlPFC activity. As with the behavioral data, the RT-enhanced FS model provided the best fit compared to all other models around the canonical hemodynamic response peak (i.e. around 7 s after transfer onset/2 s after punishment onset). The vertical black line indicates the time point when the transfer from the proposer to the responder was revealed, the light gray area indicates the transfer phase, and the darker gray area indicates the punishment window.

For the behavioral NLS procedure, we modeled punishment amount selected by our participants, in line with the assumption that the amount of punishment would be related to the utility of the responder (Wright *et al.*, 2011; Chang and Sanfey, 2013; Báez-Mendoza *et al.*, 2016). Since the majority of our participants engaged in AI-seeking behavior, we first sought to demonstrate that the original constrained version of the Fehr–Schmidt model (Standard FS) performed poorly in our task. As expected, this was the case (Figure 3). In contrast, we found that the unconstrained version of the Fehr–Schmidt model (Unconstrained FS) fitted punishment behavior much better, in fact better than all the other models without RT enhancement, including the Charness–Rabin model (Figure 3A). Thus, relaxing the constraints of Standard FS appears to enhance model fit over and above that of component and competitor models.

**Fig. 4.**
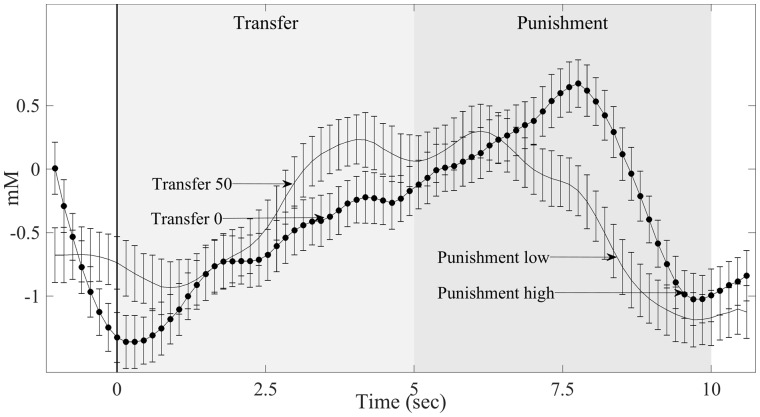
Condition-specific neural example data. Event-triggered hemodynamic dlPFC response from one participant. Data are shown separately for minimum (0) and maximum (50) transfers. Minimum transfer subsequently elicited stronger dlPFC activity and higher punishment than maximum transfer. Data are averaged across trials and aligned to the onset of the transfer phase (vertical black line). Error bars indicate SEM.

Yet, enhancing Unconstrained FS with RT as an additional parameter (RT-enhanced FS) resulted in an even better fit. This was confirmed by the nested *F*-test between Unconstrained FS and RT-enhanced FS (*F*_1_ = 77.19, *P* < 0.0001). Conversely, social preferences also clearly played a role as evidenced by a better fit of RT-enhanced FS compared to RT-only (*F*_1_ = 317.05, *P* < 0.0001). These findings confirm our hypothesis that taking RT into account improves model fit (Fischbacher *et al.*, 2013; Báez-Mendoza *et al.*, 2016) but show also that punishment behavior did not only reflect general cognitive demand.

To quantify our behavioral findings, we examined the MSEs as a measure of the distance between the fitted punishment behavior and the nine models. The RT-enhanced Fehr–Schmidt model produced a smaller mean MSE (RT-enhanced FS = 0.21) than the other models (one-way ANOVA main effect *F*_8_ = 39.583, *P* < 0.001, all Bonferroni post-hoc comparisons including the null-model *P* < 0.001; Unconstrained FS = 0.45, Standard FS = 761.37, Total Reward = 0.73, Reward Difference = 0.80, Proposer Reward = 0.69, Linear combination = 0.58, Charness–Rabin = 0.67, RT-only = 0.92). These results support the observation that the unconstrained RT-enhanced Fehr–Schmidt model explained behavior better than the other models. Thus, relaxing the constraints allows the Fehr–Schmidt model to account for AI seeking behavior observed here (see above and below) and RTs carry additional power for explaining punishment behavior in our modified ultimatum game.

### Neural model fit

Neural activity in dlPFC was sensitive to transfer amount (Figure 4 for an example). We assessed whether dlPFC activity could be explained by our social preference models using multiple linear regression. As with the behavioral data, we assessed each model’s goodness of fit using AIC and found that the Fehr–Schmidt model (Unconstrained FS) fitted the data better than all of the other models without RT, including the Charness–Rabin model (Figure 3B). Again, comparing RT-enhanced FS with Unconstrained FS or RT-only models revealed that RT as additional parameter provided an even better fit as confirmed by a significant nested *F*-test (Unconstrained FS *F*_1_ = 3.61, *P* = 0.013). Moreover, a nested *F*-test also showed that RTs alone explained dlPFC activity less well than the full model (RT-only *F*_1_ = 9.12, *P* < 0.001).

To corroborate these findings, we again examined the MSEs. As with the behavioral data, the unconstrained Fehr–Schmidt model with RT produced the smallest MSEs (RT-enhanced FS = 0.71), with the other models generating higher MSEs (one-way ANOVA main effect *F*_8_ = 24.523, *P* < 0.001, all Bonferroni post-hoc comparisons including the null-model *P* < 0.001; Unconstrained FS = 0.73, Total Reward = 0.81, Reward Difference = 0.84, Proposer Reward = 0.81, Linear Combination = 0.80, Charness–Rabin = 0.76, RT-only = 0.81). Thus, in-line with the behavioral data, the unconstrained RT-enhanced Fehr–Schmidt model outperformed the other models in terms of predictive power. Moreover, the fact that Charness–Rabin and Unconstrained FS fitted the data better than RT-only shows that including social preference terms explains dlPFC activity better than choice difficulty alone.

As mentioned above, the results primarily focused on dlPFC. It could be argued that dlPFC is particularly sensitive to RT and that a pure social utility model without RT would fit dmPFC activity better than dlPFC activity. A comparison of the MSEs between dlPFC and dmPFC revealed no difference in model fit as assessed using *t*-test, for the Unconstrained FS model (*P* = 0.432) or for Charness–Rabin (*P* = 0.612). Thus, our data provide no evidence to suggest that dmPFC more closely captures RT-independent social utility than dlPFC activity (see also section dlPFC predicts behavior better than dmPFC).

Considering the 40 sliding time intervals, the unconstrained RT-enhanced Fehr–Schmidt model fitted the data best around the typical canonical hemodynamic response peak (which is assumed to occur at 5–7 s after stimulus onset, as reported by previous fNIRS studies—Jasdzewski *et al.*, 2003; Matthews *et al.*, 2008; Cui *et al.*, 2010; Hoshi, 2011). In other words, the best fit was around seven seconds after transfer onset, i.e. around 2 s after punishment onset (Figure 3B). Given the typical peak times, these data suggest that the responses were driven primarily by the transfer period.

### Social preference parameters in behavior and brain

Next, we investigated the parameters capturing social preference towards AI and DI as obtained from the behavioral and neural fits to the RT-enhanced FS model in more detail (note that this model was the best-fitting model in both domains). The distributions of the α parameter (DI) ranged from −0.033 to 0.153 (behavioral) and −0.058 to 0.127 (neural), those of the β parameter (AI) from −1 to 0 (behavioral) and −1 to 0.045 (neural). Thus, participants showed considerable variation in their behavioral and neural valuation of inequality. Moreover, given that β parameters were predominantly negative, participants appeared to be more AI-seeking than AI-averse in the present paradigm.

The estimates from the quadratic regression between RT and transfer correlated positively with participants’ α parameters (DI) (*r*** **= 0.369, *P*** **= 0.011) but not with the β parameter (AI) (*r*** **= 0.104, *P*** **= 0.479), suggesting that participants with stronger DI aversion responded relatively more slowly to intermediate offers. Thus, participants with lower DI aversion appear to be less sensitive to the inequalities arising from intermediate transfer amounts and therefore require less time to generate a punishment in these situations.

**Fig. 5.**
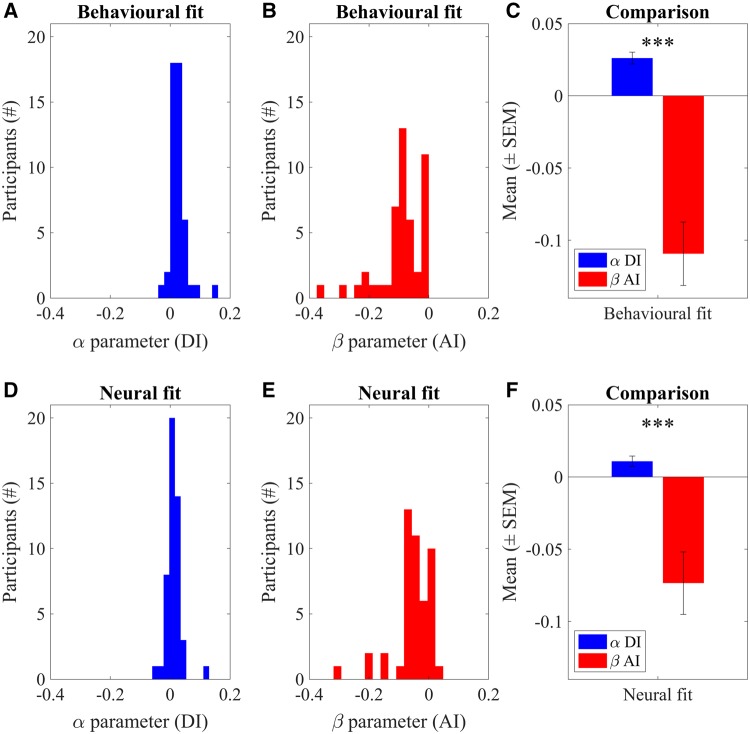
Preference parameters. Distributions of (A, B) the behavioral and (D, E) the neural participant-specific best fitting preference parameters (α for DI, β for AI) according to the RT-enhanced Fehr–Schmidt model. The positive weight on DI reflects DI aversion whereas the negative weight on AI reflects AI-seeking. Note that one outlier has been excluded from the figures in A and D. (C, F) Comparison of the distribution of the participant-specific social preference parameters for the behavioral and neural model fits (based on A, B). There were no significant differences between the behavioral and neural model fits. Error bars indicate standard error of the mean (SEM). See Table 2 for statistics.

When we directly compared the preference parameters for AI and DI, we found a significant difference between them irrespective of whether the data were behavioral or neural (two-way ANOVA, main effect of ‘model parameters’ *F*_3_** **= 38.05, *P*** **< 0.001; main effect of ‘data type’ *F*_1_** **= 1.88, *P*** **= 0.171; interaction effect ‘data type × model parameters’ *F*_3_** **= 2.11, *P*** **= 0.099, Table 2). The α parameters (DI) were significantly larger (more positive) compared to the (more negative) β parameters (AI) for both the behavioral and neural fits (Figure 5) (post-hoc comparison assessing α** **> β group-level: behavioral and neural fits both *P*** **< 0.001; single-participant level: behavioral fit 88%, neural fit 98%). Thus, our participants showed significantly greater aversion to DI than AI and thereby satisfied the constraint α** **≥ β of the original Fehr–Schmidt model at both the behavioral and neural level.

**Table 2.** Comparison of behavioral fit *vs* neural fit

		Unconstrained FS	RT-enhanced FS
**Main effects**	**Data type**	*F*_1_ = 0.234, *P* = 0.629	*F*_1_ = 1.881, *P* = 0.171
	**Model parameters**	*F*_3_ = 46.092, *P* < 0.001	*F*_3_ = 38.046, *P* < 0.001
	**Data type × model parameters**	*F*_3_ = 0.024, *P* = 0.976	*F*_3_ = 2.107, *P* = 0.099
**Post-hoc comparisons**		***P*-value**	***P*-value**
	Neural DI *vs* Neural AI	<0.0001	<0.0001
	Neural DI *vs* Behavioral DI	0.999	0.979
	Neural AI *vs* Behavioral AI	1.000	0.309
	Behavioral DI *vs* Behavioral AI	<0.0001	<0.0001

Social preference parameters were compared using two-way ANOVA with the within-subject factors ‘data type’ (behavioral fit *vs* neural fit) and ‘model parameters’ (DI, i.e. α; AI, i.e. β). Bonferroni correction was used to account for multiple post-hoc comparisons. See Figure 5 for illustration.

By extension, the relatively poor fit of the original, constrained Fehr–Schmidt model should be explained by a failure to fulfill the constraint that people have to be AI-neutral or AI-averse (0** **≤ β). Indeed, post-hoc comparisons showed that β was significantly smaller than 0 (group-level: behavioral and neural fits both, *P*** **< 0.001; single-participant level: behavioral fit 77%, neural fit 79%). In other words, on average our responders were only averse to being worse off but exhibited a preference for being better off than the proposer, which was reflected in both behavioral and neural fits. Together, our findings indicated that responders not only exhibited aversion for DI (as expected), but that a number of responders also exhibited a clear preference for being better off than the proposer. Thus, in our experimental setup, social preferences went beyond the motive of minimizing objective payoff inequality.

We also compared the subgroup of participants with AI-seeking behavior (38 out of 48) to those without such behavior. As expected, AI-seeking participants had significantly more negative β parameters (AI) (behavioral fit *F*** **= 7.40, *P*** **= 0.009; hemodynamic fit *F*** **= 4.06, *P*** **= 0.049) but no differences in α parameters (DI) (behavioral fit *F*** **= 3.34, *P*** **= 0.074; hemodynamic fit *F*** **= 0.22, *P*** **= 0.645). AI-seeking participants also showed significantly longer RTs (behavioral fit *F*** **= 14.18, *P*** **= 0.0005; hemodynamic fit *F*** **= 20.49, *P*** **< 0.0001) and selected larger punishment amounts (behavioral fit *F*** **= 48.25, *P*** **< 0.0001; hemodynamic fit *F*** **= 38.38, *P*** **< 0.0001). Note that not only the punishment differences but also the RT differences are expected as achieving AI in our task requires punishing more, which requires larger movements, resulting in longer RTs.

### Psychometric-neurometric correlations

**Fig. 6.**
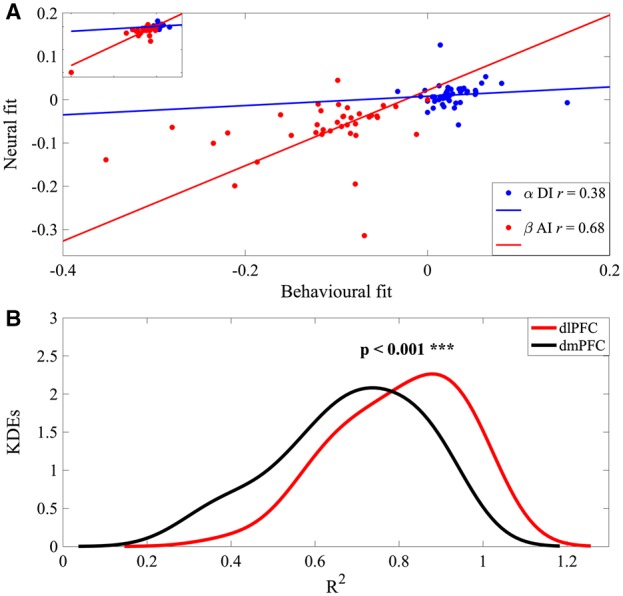
Psychometric–neurometric correlation and prediction. (A) Correlation coefficients (*r*) of percentage-bend correlation (Pernet *et al.*, 2012) between the participant-specific best fitting model parameters (α for DI, β for AI). Model parameters are from the RT-enhanced Fehr–Schmidt model as applied to punishment behavior and dlPFC activity. Inset (top left) shows full data set, including one outlier, which was excluded in main figure. (B) Prediction. Kernel density estimates (KDEs) of the coefficients of determination (*R*^2^) for the trial-based prediction of the behavioral fit based on the neural fit (dlPFC and dmPFC) according to the RT-enhanced Fehr–Schmidt model. There was a significant difference between dlPFC and dmPFC **(***P* < 0.001**;** indicated by ***. See Table 3 for statistics**).**

We next asked whether there would be significant differences in how intimately dlPFC activity relates to different preference parameters obtained from the behavioral and the neural fits (psychometric–neurometric relation). In particular, we used percentage-bend correlation (Pernet *et al.*, 2012) as a robust method that protects against outliers among marginal distributions (which were present in our heterogeneous distribution of subject-specific model parameters). Parameter-wise correlations were positive and significant for both DI (α parameter *r*** **= 0.38, *P*** **= 0.007) and AI (β parameter *r*** **= 0.70, *P*** **< 0.001; Figure 6A), in-line with a social utility signal in dlPFC. Moreover, the behavioral–neural correlation was significantly higher for the β parameter than for the α parameter (*P*** **= 0.027 Fisher’s *r*-to-*z* transformation followed by *t*-test) (Figure 6A). These findings indicate that dlPFC activity preferentially relates to subjective attitudes toward AI rather than DI.

### dlPFC predicts behavior better than dmPFC

Finally, we compared dlPFC with dmPFC data with regard to how strongly they represent social preferences. Specifically, we performed a prediction analysis to assess whether the neural responses in the dlPFC and dmPFC would predict behavioral punishment differently. To test this possibility, we conducted linear support vector machine (SVM) regression per participant with utility (as calculated according to Equation 8) based on the neural fit (predictor variable) and the behavioral fit (dependent variable). We cross-validated the linear SVM regression by specifying a 50% holdout sample for testing and training the model. For each participant, we selected the largest coefficients of determination (*R*^2^) within the punishment window (which approximately corresponded to the peak of the model fit, see Figure 3B) and illustrated their probability density estimates on the group-level (Figure 6B). As may be expected based on the psychometric–neurometric correlations reported above, we found that the neural fit in dlPFC based on the RT-enhanced Fehr–Schmidt model strongly predicted the behavioral fit (mean *R*^2^** **= 0.80, range 0.40–0.99). In contrast, performing the same prediction for the dmPFC revealed that the predictive capability of the neural fit was significantly smaller (mean *R*^2^** **= 0.68, range 0.29–0.92) as assessed using paired *t*-test (*P*** **< 0.001, Table 3). These findings suggest that the subjective responses to transfers were more closely associated with neural responses in the dlPFC than the dmPFC. By extension, the dlPFC appears to be more strongly involved in representing behavioral social preferences than the dmPFC in our task.

**Table 3.** Differential prediction of transfer behavior

	dlPFC Unconstrained FS	dmPFC Unconstrained FS	dlPFC RT-enhanced FS	dmPFC RT-enhanced FS
	(*R*^2^=0.68)	(*R*^2^=0.59)	(*R*^2^=0.80)	(*R*^2^=0.68)
dlPFC Unconstrained FS		0.000005	0.000009	0.356297
dmPFC Unconstrained FS	0.000005		<0.000001	0.003826
dlPFC RT-enhanced FS	0.000009	<0.000001		0.000014
dmPFC RT-enhanced FS	0.356297	0.003826	0.000014	

*T*-test comparing the coefficients of determination (R2) for the prediction of the neural fit (dlPFC and dmPFC) based on the behavioral fit according to the unconstrained Fehr-Schmidt model without and with RT (Unconstrained FS and RT-enhanced FS). See Figure 6B for illustration. Predictions for dlPFC RT-enhanced FS revelead the highest R2 followed by dmPFC RT-enhanced FS, dlPFC and dmPFC Unconstrained FS.

## Discussion

This study investigated preferences toward DI and AI at the behavioral and the neural level. In our task, both behavioral and neural responses were better explained by an unconstrained variant of the Fehr–Schmidt model compared to the constrained Fehr–Schmidt model, the Charness–Rabin model or any of the simpler models that considered constituent terms. Accordingly, our data reveal AI seeking and a social utility signal in dlPFC. Moreover, they provide important clues for how changes to the standard Fehr–Schmidt model could increase its ability to model the neural implementation of social preferences in dlPFC, by taking into account the impact of executive functions, decision difficulty or working memory. In particular, including RT further enhanced model fit, similar to findings in non-human primates (Báez-Mendoza *et al.*, 2016), indicating that dlPFC activity is modulated by both inequality and executive functions, such as working memory load (Curtis and D’Esposito, 2003). Finally, by recording from two areas simultaneously, our data suggest a tighter link between the behavioral expression of social preferences and dlPFC activity as compared to dmPFC activity.

The activity in dlPFC was well explained by an unconstrained RT-enhanced Fehr–Schmidt model. Our study thereby suggests that the dlPFC processes a social utility signal and that this role of dlPFC is not limited to implementing fairness norms (Knoch *et al.*, 2006). Moreover, it explains and qualifies previously reported dlPFC involvement in inequality paradigms (Chang *et al.*, 2011; Fliessbach *et al.*, 2012; Güroğlu *et al.*, 2014; Nihonsugi *et al.*, 2015). For example, while significantly stronger coding of DI than AI has been shown previously in a situation in which participants could not influence relative payoffs (Fliessbach *et al.*, 2012), our data in addition reveal that in a situation where relative payoffs can be changed, the relation between neural and behavioral coding of model-based social preferences is significantly stronger for AI than DI (Figure 6A). Thus, the social utility signal in dlPFC appears to be sensitive to instrumental contingencies and behavioral relevance.

The original Fehr–Schmidt model (1999) assumes that aversion against DI would be greater than that against AI (i.e. α** **≥ β). Second, the model assumes that aversion against AI ought to be larger than or equal to 0 (0** **≤ β), presuming that participants prefer equality to being better off than others. The standard model has been criticized for being based on paradigms that allow only for a limited range of social preferences (Charness and Rabin, 2002) and the assumptions for being based on games with a limited number of available actions (Binmore and Shaked, 2010a,b; Fehr and Schmidt, 2010). In our paradigm, the possibility to punish proposers by a self-determined amount and thereby create AI, E or DI provided participants with a larger number of available actions than simply rejecting or accepting proposed transfers. However, our approach also suffers from limitations. By excluding transfers that would have created AI rather than *E* or DI for the responder it was not possible to behaviorally determine AI aversion. Note though that AI-creating transfers would have been somewhat unrealistic because they rarely occur and because transfers that create *E* are sufficient to ensure acceptance in the standard ultimatum game (Fehr and Schmidt, 1999). Another limitation is that we allowed participants only to subtract rather than add to the payoff of the proposer. Therefore, the degree of punishment was the focal point of the task, which may have contributed to higher-than-usual levels of AI-seeking. Finally, we may have biased our participants into AI-seeking behavior through the strong impact of punishment on proposer payoff. In any case, the original Fehr–Schmidt model is nested within our unconstrained version, meaning that the unconstrained model will describe non-conventional social preferences in a larger variety of situations.

While the first constraint of the original Fehr-Schmidt model (i.e. that α** **≥ β) was met (i.e. participants were more averse to DI than AI), the second assumption (i.e. that 0** **≤ β) was not. The procedure of relaxing the constraints of the original model is in line with earlier studies reporting that β can be larger than α (Bellemare *et al.*, 2008), and others estimating negative α values in some conditions (Loewenstein *et al.*, 1989; Hoppe and Schmitz, 2013) or individuals (Fliessbach *et al.*, 2012). The unconstrained model thus may allow for a more realistic picture of participant- and situation-specific social preferences and associated individual neural responses to economic inequality.

AI-seeking behavior left both players worse off in our task compared to not punishing at all, which contradicts both AI aversion as assumed by the original Fehr–Schmidt model and efficiency maximization of the Charness–Rabin model. While the standard version of the Fehr–Schmidt model performed worse than the Charness–Rabin model, the unconstrained version performed better. Hence, by allowing for AI-seeking the unconstrained model can account for a wider range of real-world and experimental behavior (Ball and Eckel, 1998; Chmura *et al.*, 2005; Vostroknutov *et al.*, 2012). Moreover, our data reinforce the notion that participants who have little or no prior knowledge of efficiency concerns put little weight on efficiency (Fehr *et al.*, 2006).

Including RT explained additional variance in punishment behavior. In line with previous research (Knoch *et al.*, 2006; Baumgartner *et al.*, 2011; Harlé and Sanfey, 2012; Knight, 2012; Ma *et al.*, 2015), we found that our participants were significantly slower in punishing somewhat unfair transfers than in accepting fair offers or punishing extremely unfair transfers. Thus, after somewhat unfair transfers, fairness norms appear to have conflicted with the desire to maximize payoff, whereas with smaller or larger transfers conflict may have been reduced. Please note that only a person who in addition to self-interest also takes social motives into account would ever experience conflict (Fischbacher *et al.*, 2013). Our data are in line with this view and more generally indicate that RT provide important information about the process underlying any kind of decisions, including social ones (Evans *et al.*, 2015; Krajbich *et al.*, 2015).

We found that incorporating RT into our model also increased model fit for dlPFC data. This may have been expected based on previous findings that related RT to dlPFC function. For example, disruption of dlPFC activity not only reduced participants’ willingness to reject unfair offers in more standard ultimatum games, but also diminished the RT difference between unfair and fair offers typically seen in participants with intact dlPFC function (Knoch *et al.*, 2006). Moreover, dlPFC activity often correlates with behavioral RT (Yarkoni *et al.*, 2009; Grinband *et al.*, 2011), possibly reflecting executive functions such as choice difficulty, cognitive control, and/or working memory load (Curtis and D’Esposito, 2003). Our findings suggest that such a relation also holds for decisions that allowed for AI-seeking, in addition to simply abolishing *vs* tolerating DI in the standard ultimatum game.

Activity in dlPFC predicted punishment behavior better than dmPFC activity. Thus, compared to dmPFC, dlPFC appears to be more sensitive to behaviorally relevant differences between the outcomes of others and own outcomes. Previous studies suggest that dmPFC may be more strongly involved in representing the preferences of others (Morelli *et al.*, 2015; Seid-Fatemi & Tobler, 2015) and in updating one’s own preferences based on social information (Izuma and Adolphs, 2013), indicating a subtle prefrontal differentiation of social value signals.

To conclude, our results suggest that dlPFC activity reflects a socially modulated value signal reflecting individual social preferences regarding payoff differences and suggest ways in which social preference models could be enhanced.
